# Optimum breeding strategies using genomic and phenotypic selection for the simultaneous improvement of two traits

**DOI:** 10.1007/s00122-021-03945-5

**Published:** 2021-10-07

**Authors:** Jose J. Marulanda, Xuefei Mi, H. Friedrich Utz, Albrecht E. Melchinger, Tobias Würschum, C. Friedrich H. Longin

**Affiliations:** 1 Institute of Plant Breeding, Seed Science and Population Genetics, University of Hohenheim, 70599 Stuttgart, Germany; 2 State Plant Breeding Institute, University of Hohenheim, 70599 Stuttgart, Germany; 3 Agricultural Genomics Institute, Chinese Academy of Agricultural Sciences, Shenzhen, 518120 China

## Abstract

****Key message**:**

** A breeding strategy combining genomic with one-stage phenotypic selection maximizes annual selection gain for net merit. Choice of the selection index strongly affects the selection gain expected in individual traits.**

**Abstract:**

Selection indices using genomic information have been proposed in crop-specific scenarios. Routine use of genomic selection (GS) for simultaneous improvement of multiple traits requires information about the impact of the available economic and logistic resources and genetic properties (variances, trait correlations, and prediction accuracies) of the breeding population on the expected selection gain. We extended the R package “*selectiongain*” from single trait to index selection to optimize and compare breeding strategies for simultaneous improvement of two traits. We focused on the expected annual selection gain (*ΔG*_*a*_*)* for traits differing in their genetic correlation, economic weights, variance components, and prediction accuracies of GS. For all scenarios considered, breeding strategy *GSrapid* (one-stage GS followed by one-stage phenotypic selection) achieved higher Δ*G*_a_ than classical two-stage phenotypic selection, regardless of the index chosen to combine the two traits and the prediction accuracy of GS. The Smith–Hazel or base index delivered higher Δ*G*_a_ for net merit and individual traits compared to selection by independent culling levels, whereas the restricted index led to lower *ΔG*_*a*_ in net merit and divergent results for selection gain of individual traits. The differences among the indices depended strongly on the correlation of traits, their variance components, and economic weights, underpinning the importance of choosing the selection indices according to the goal of the breeding program. We demonstrate our theoretical derivations and extensions of the R package “*selectiongain*” with an example from hybrid wheat by designing indices to simultaneously improve grain yield and grain protein content or sedimentation volume.

**Supplementary Information:**

The online version contains supplementary material available at 10.1007/s00122-021-03945-5.

## Introduction

Plant breeding aims at the improvement of the economic merit of cultivars, which depends on the performance of the traits composing the product profile or variety concept (Bernardo 2010). Attempts to combine information from several sources in one single value (net merit) date back to Galton´s “Law of Regression”, which was presented even before the Mendelian nature of inheritance was clear (Hazel 1943). In their classical formulation, selection indices are linear functions of the target traits, each of them receiving a certain weight depending on its importance. Currently, index selection is a key tool in animal breeding programs as it facilitates the process of making consistent breeding decisions across years (Cole and VanRaden 2017). In plant breeding, to select for single traits using independent culling levels (ICL) is a widely adopted practice as phenotypic data for all traits are not readily available or not affordable to be screened at a large scale. However, selection indices have been shown to achieve more significant and more consistent genetic gains than ICL (Hazel 1943), exerting major value in the population improvement phase of a plant breeding program (Gaynor et al. 2017). Widely used indices as the Smith–Hazel index (Hazel 1943), base index (Williams 1962), and restricted index (Kempthorne and Nordskog 1959) are constructed with (1) phenotypic and genetic correlations, (2) variances and covariances between traits, and (3) the economic importance assigned to each trait. The Smith–Hazel index (SH) behaves as an “optimum index” when variances and covariances are obtained without error. As this is rarely the case, the base index uses only the economic importance of each trait as the weight and disregard phenotypic and genotypic covariances. The restricted index of Kempthorne and Nordskog uses variance and covariance components and economic importance to improve one trait while keeping the second constant. Despite these three indices requiring knowledge on each trait's economic importance, determining these economic weights is a cumbersome process in plant breeding (Mistele et al 1994). Breeding for multiple traits has proven challenging when negative correlations exist between traits (Bernardo 2010). Well-known examples for negatively correlated traits are grain yield and protein content in bread wheat (Longin et al. 2013a; Laidig et al. 2016) and durum wheat (Longin et al. 2013b; Rapp et al. 2018), and dry matter yield and dry matter content in maize (Grieder et al. 2012).

Genomic selection opened new avenues to implement simultaneous selection for several traits, with index selection being a routine practice in plant breeding. Currently, wheat and maize breeding programs use phenotypic data from previous years to build large calibration sets. These enable practitioners to simultaneously access genomic estimated breeding values (GEBV) of all required traits needed for index selection. Several theoretical and practical attempts to generate a selection index using information from GEBV and to compute the realized response to selection have been made (Dekkers 2007; Ceron-Rojas et al. 2015; Michel et al. 2019). However, studies calculating the expected selection gain (*ΔG)* and optimum allocation of resources in breeding programs for index selection are lacking in the literature. In particular, the influence of different indices on the ranking of breeding strategies and their optimum allocation of resources when simultaneously improving traits with contrasting economic relevance, levels of genetic correlation, and prediction accuracy in genomic selection (GS) for the same budget and logistical constraints is of major relevance in practical plant breeding. Further studies are required to estimate the decrease in selection gain for the individual traits when moving from model calculations for single-trait improvement to multiple-trait improvement via ICL or index selection. For a single trait, implementation of GS in plant breeding strategies has shown promising results based on model calculations. A breeding strategy with one-stage GS followed by one-stage phenotypic selection is expected to increase the annual selection gain for hybrid and line breeding in wheat by more than 30% compared with classical two-stage phenotypic selection (Longin et al. 2015) and similar improvements are expected in the annual selection gain for other important crops like maize, rye, triticale, and barley (Marulanda et al. 2016). However, further research is warranted for implementing GS in plant breeding programs aiming to improve several traits simultaneously. Therefore, we expanded the open-source R package *“selectiongain”* (Mi et al. 2014) to estimate selection gain and optimize the allocation of resources for index selection to simultaneously improve two traits at the population improvement phase of a breeding program. Our objectives were to (1) determine how the correlation between traits, the economic weights, and the variance components affect selection gain of the net merit and each trait separately, (2) examine the consequences of the choice of different selection indices in breeding strategies including genomic and phenotypic selection, and (3) identify efficient breeding strategies to simultaneously improve traits with different prediction accuracies. Finally, we study a specific case for negatively correlated traits in hybrid wheat in the practical section.

## Materials and methods

### Breeding strategies

We compared two breeding strategies for hybrid breeding, a classical two-stage phenotypic selection (*PSstandard*) and a breeding scheme using one-stage genomic selection followed by one-stage phenotypic selection (*GSrapid*). Both strategies, originally proposed by Longin et al. (2015) and modified by Marulanda et al. (2016), start with the production of *N*_*ini*_ doubled haploid (DH) lines from crosses of numerous DH parents and finish with the selection of the five DH lines with highest general combining ability (GCA; Suppl. Figure 1). First, the *N*_*ini*_ DH lines are tested (and multiplied) in an observation nursery for their line per se performance regarding highly heritable traits, which are not correlated with the net merit (index for the two target traits). According to the conclusions of Marulanda et al. (2016), after this nursery assessment, we assumed that only 25% of the lines are selected and promoted to evaluation in one or two stages of field tests for GCA. The first strategy, *GSrapid,* includes a selection stage for GCA of net merit based on the GEBV of the *N*_*1*_ DH lines selected in the nursery. After GS, one stage of phenotypic selection for GCA of net merit with *N*_*2*_ candidates is performed. The second strategy, *PSstandard,* represents the conventional practice in hybrid cereal breeding programs with two stages of phenotypic selection and was considered as benchmark. For hybrid seed production, we assumed the use of a chemical hybridization agent (CHA), and hybrids were tested in multi-environment trials conducted at *L*_*j*_ locations with *T*_*j*_ inbred testers in selection stage two and three (*j* = 2, 3). Without restrictions on *L*_*j*_, selection gain is maximized by one replication per location (Melchinger et al. 2005). Thus, we set the number of replications to one for all calculations.

### Optimum allocation of resources and calculation of selection gain

Calculation of the selection gain was based on the well-known formula of Cochran (1951) with multivariate normal integrals for selected fractions and heritabilities. As all our results were generated with these model calculations, we present strictly speaking the expected selection gain for the investigated scenarios. For sake of simplicity, however, we disregard the term “expected” in the following. As the investigated breeding strategies differ in their cycle length, we further determined the annual selection gain, which is the absolute expected selection gain divided by the number of years required to finish one breeding cycle (Suppl. Figure 1).

A vector [*N*_*ini*_, *N*_*1*_, *N*_*j*_, *L*_*j*_, *T*_*j*_] represents the number of DH lines, testers, and locations at the initial stage (*ini*) or stage *j*. This vector corresponds to the optimum allocation only if it maximizes selection gain in the respective breeding strategy for a given scenario regarding variance components, budgets, and costs. The optimum allocation was determined by a grid search across all possible allocations for the scenario under consideration (for details see Mi et al. 2015). Different breeding strategies were only compared at their respective optimum allocation for the scenario under consideration. Thus, all our results and comparisons are based on the maximum annual selection gain, which is in the following referred to as *ΔG*_*a*_. We also present the annual selection gain for the individual traits when the allocation of resources is optimized for the net merit. For comparison, optimization of strategies for single-trait improvement was performed to estimate the maximum annual selection gain for trait 1 and trait 2. For all our calculations, we used the open-source R (R Core Team 2016) package “*selectiongain*”, which was extended for the incorporation of index selection including phenotypic and genomic information for two traits. For details about the package, the reader is referred to Mi et al. (2015) and the user manual of version 2.0.65 (http://cran.r-project.org/web/packages/selectiongain/selectiongain.pdf).

### Quantitative genetic parameters and economic frame

This manuscript is divided into two sections. First, we investigated scenarios with two hypothetical traits to assess the influence of three parameters (correlation of traits, variance components, and economic weights) on the optimum allocation of test resources and *ΔG*_*a*_ under four different indices and two breeding schemes. We defined a standard scenario assuming that both traits have equal variance components, equal economic weights (each trait weight was set to 1, so the sum of weights equals 2), and a genetic correlation between traits of -0.5 (scenario I, Table 1). As variance components, we used reported values for hybrid wheat (Longin et al. 2013a) and standardized them to a GCA variance of 1. Afterward, possible effects of each of the three parameters were assessed under *ceteris paribus* conditions, i.e., varying only one parameter at a time and comparing the results with the standard scenario. Further, scenarios compared to the standard scenario were: a genetic correlation between both traits of 0 and 0.5 (scenarios II and III, respectively), an economic weight of 0 for trait two (trait one weight 2, scenario IV), and economic weight of 4/3 for trait two, so that it has twice the economic importance of trait one (scenario V). As defined by Osthushenrich et al. (2018), the phenotypic variance equals the sum of GCA and masking variance, which is due to interaction effects (e.g., genotype by environment) and the residual error. For investigating the influence of variance components, we compared the standard scenario (equal variance components for both traits) with scenarios, where the GCA variance of trait two was three times higher than the GCA variance of trait 1 (scenario VI), or where the masking variance for trait 2 was three times higher than the masking variance of trait one (scenario VII; Table 1). In the second section, we extended our calculations to hybrid wheat breeding with alternative goals: (1) improve simultaneously grain yield and protein content and (2) improve simultaneously grain yield and sedimentation volume based on empirical data on variance components, correlation of traits, and economic weights as explained below.

**Table 1:** Scenarios investigated on the first section of this study. Parameters differing from those in the standard scenario I are given in boldface

Parameters under study										
Scenario	*ρ*_Genetic_	Economic weight				Variance components for trait 2				
		Trait 1		Trait 2		${\sigma }_{GCA}^{2}$	${\sigma }_{GCA\times L}^{2}$	${\sigma }_{SCA}^{2}$	${\sigma }_{SCA\times L}^{2}$	${\sigma }_{error}^{2}$
I	− 0.5	1		1		1	0.91	0.33	0.52	4.28
II	**0**	1		1		1	0.91	0.33	0.52	4.28
III	**0.5**	1		1		1	0.91	0.33	0.52	4.28
IV	− 0.5	**2**		**0**		1	0.91	0.33	0.52	4.28
V	− 0.5	**0.7**		**1.3**		1	0.91	0.33	0.52	4.28
VI	− 0.5	1		1		**1 × 3**	0.91	0.33	0.52	4.28
VII	− 0.5	1		1		1	**0.91 × 3**	**0.33 × 3**	**0.52 × 3**	**4.28 × 3**

Variance components of trait 1 are always equal to ${\sigma }_{GCA}^{2}=1$, ${\sigma }_{GCA \times L}^{2}=0.91$, ${\sigma }_{SCA}^{2}=0.33$, ${\sigma }_{SCA \times L}^{2}=0.52$, and ${\sigma }_{error}^{2}=4.28$

Other assumptions required for the model calculations were taken from the literature (cf. Marulanda et al. 2016). These assumptions include: a fixed total budget of 10,000 field plot equivalents was available for the following activities: (1) producing DH lines and testcross seed, (2) evaluating line per se performance in the observation nursery, and (3) genotyping and assessing testcross performance in the field trials. The following costs, expressed as field plot equivalents, were assumed: *Cost*_*DH*_ = 1, *Cost*_*Genotyping*_ = 2, and *Cost*_*Hybridseed*_ = 4. The assessment of protein content (through near-infrared spectroscopy, NIRS, ICC standard method 159, ICC, Austria) and sedimentation volume according to Zeleny (ICC standard method 116/1, ICC, Austria) was assumed to be already included in the cost of field assessment. Typically, one stage of hybrid seed production delivers enough seeds for two years of phenotyping.

Before routinely using GS, calibration experiments are required to develop prediction models. We assumed that GS calibrations were already developed outside the breeding cycle. Furthermore, recalibrations of models are regularly needed in the breeding program (Heslot et al. 2015), but these can be done with data available from routine breeding without additional budget requirements. The genetic architecture of the trait, the method of assessment, and the environmental effects could lead to different levels of GS accuracy for different traits (cf. Zhao et al. 2013; Liu et al. 2016). We, therefore, varied the prediction accuracies from 0.1 to 0.7, increasing by intervals of 0.1, and compared two assumptions: (1) *“equal prediction accuracies”*: both traits are assumed to have equal prediction accuracies and (2) *“unequal prediction accuracies”*: the prediction accuracy of trait 2 is always 0.2 higher than the accuracy of trait 1. Simultaneous testing of unequal prediction accuracies and economic weights or variance components results in numerous scenarios, e.g., trait 2 has a higher prediction accuracy than trait 1 and all economic weights. To reduce the complexity in the manuscript, we intentionally left some comparisons out of our study. However, the readers can find good approximations for scenarios not included when exchanging the conclusions for the traits in the scenarios presented. For instance, scenario V approaches the example mentioned above as trait 2 has larger prediction accuracy than trait 1 and two times the economic weight of trait 1.

We used variance and covariance components of a recent large experimental study on hybrid wheat (Liu et al. 2016). For the theoretical section, the variance components for grain yield (dt h^−1^) were used as a reference and both traits were assumed to have equal variance components as follows: ${\sigma }_{GCA}^{2}=5.7$, ${\sigma }_{GCA \times L}^{2}=5.19$, ${\sigma }_{SCA}^{2}=1.88$, ${\sigma }_{SCA \times L}^{2}=2.94$, and ${\sigma }_{error}^{2}=24.37$. For the practical section on hybrid wheat, we used these variance components of grain yield for trait 1 and the following estimates of protein content (%) for trait 2: ${\sigma }_{GCA}^{2}=0.08$, ${\sigma }_{GCA \times L}^{2}=0.02$, ${\sigma }_{SCA}^{2}=0.01$, ${\sigma }_{SCA \times L}^{2}=0.00$ and ${\sigma }_{error}^{2}=0.09$ or sedimentation volume (ml) ${\sigma }_{GCA}^{2}=23.54$, ${\sigma }_{GCA \times L}^{2}=1.58$, ${\sigma }_{SCA}^{2}=0.64$, ${\sigma }_{SCA \times L}^{2}=0.25$ and ${\sigma }_{error}^{2}=6.9$ (Goals 1 and 2, respectively). Further information can be found in the Supp. Table. 1. The genetic correlations were − 0.40 for grain yield and protein content and − 0.18 for grain yield and sedimentation volume (Zhao, pers. comm.). For simplicity, and as most breeders have experimental data available, we assumed that the covariances between both traits were zero for their specific combining ability (SCA) effects, their GCA x location, and SCA x location interactions. To verify whether these components have a high impact on the outcome of our model calculations, we additionally determined the optimum allocation and *ΔG*_*a*_ for intermediate and high values for these covariance components (data not shown).

Economic weights in the practical section were determined from the marginal return obtained by farmers in the German market as described by Laidig et al. (2016). Briefly, a farmer producing fodder quality wheat (C-grade), received 17.23 € per dt. Higher quality was remunerated by extra payments of 1.0 € per dt for B quality wheat, 2.0 € per dt for A quality wheat, and 4.5 € per dt for E quality wheat compared to C quality. While B class grain is used directly for bread-making purposes, the A and E classes are used for blending to obtain a specific raw bakery material. Based on these values, we reported the economic weights as relative values to the price per dt of 17.23. So, the economic weight for grain yield is set to 1 and the weight of grain protein content or sedimentation volume corresponds to the increase for quality grade A (2/17.23 = 0.12). The weight of this category is closest to the average of the increase in the four quality categories (0.11).

### Implementation of index selection

The proposed implementation of index selection to deterministically evaluate and optimize the use of genomic selection for multiple traits in hybrid breeding programs was inspired by combining ideas from three main publications. While Utz (1969) provided estimates of covariance for traits in multiple-stage selection, Wricke and Weber (1986) explained in detail the construction of indices in multiple-stage selection and Dekkers (2007) gave an extension to multiple-trait marker-assisted and phenotypic selection. We considered a group of $m$ traits for which phenotypic scores are available and denoted as ${x}_{j}$ ($j=1,\dots , m$). The second group of traits corresponds to breeding values ${y}_{l}$ ($l =1,\dots , n),$ which are improved by selection on ${x}_{j}$. Traits may overlap in both groups.

We constructed indices for both groups: first a phenotypic index $I=\sum_{j=1}^{m}{b}_{j}{x}_{j}$ and second a genotypic index $=\sum_{l=1}^{n}{a}_{l}{y}_{l}$. The genotypic index, known as the net merit, corresponds to the criterion for which we want to maximize the response to selection. The economic weights ${a}_{l}$ can be (1) chosen by the breeder according to her/his experience, (2) derived from the monetary returns produced by each trait, or (3) determined from selection decisions made in previous years in the breeding program. The phenotypic weights ${b}_{j}$ should be chosen such that selection based on the phenotypic traits ${x}_{j}$ maximizes the response to selection for net merit$H$. The selection gain *ΔG* for the net merit $H$ can be estimated by$=i\frac{{cov}_{(I,H)} }{{\sigma }_{I}}$. In the frame of multiple-stage selection, the selection intensity $i$ can be determined using the multivariate normal distribution as implemented in the package “*selectiongain*”. In this study, we expanded the function *multistagecor* of *“selectiongain*” to estimate the covariance ${cov}_{(I,H)}$ between the phenotypic index and the net merit and the variance ${\sigma }_{I}^{2}$ of the phenotypic index as presented in detail in the appendix. The new version of the R package “selectiongain” (2.0.65) is online available, including the open-source code of the functions to be used by the scientific community. Additionally, we have included supplementary file 1 (R script) in which we use the new version of the package *“selectiongain*” to solve two numerical examples. First, we solve the numerical example of Wricke and Weber (1986) for the selection of two traits in one stage using the Smith–Hazel index (chapter 12 page 348). Second, we estimate the expected genetic gain in a multistage selection scenario when two traits are improved using the Smith–Hazel index. We used the input parameters of the scenario standard and provide the expected genetic gain after each selection stage for each trait. Finally, we introduced references to the formulas presented in the appendix, facilitating the comprehension of the step-by-step process to compute the expected genetic gain.

Indices proposed in literature include the Smith–Hazel index (Hazel 1943), the base index (Williams 1962), and the restricted index (Kempthorne and Nordskog 1959). While these indices are linear functions of several traits, they vary on the assignment of weights to each trait. Smith–Hazel index uses information from the phenotypic and genotypic covariances, and the economic importance to calculate the weights. In the rare case of having estimates of variances and covariances without error, this index becomes the “optimum index”. The base index uses the economic importance of each trait directly as the weight, neglecting phenotypic and genotypic covariances. The restricted index uses variance and covariance components and economic importance to improve one trait while keeping the second constant. For further details, the reader is referred to the appendix.

We used two benchmarks to assess the impact on *ΔG*_*a*_ of both the net merit and individual traits when using index selection. First, we used single-trait selection as benchmark to estimate the reduction in *ΔG*_*a*_ when moving from a strategy to improve only one trait to a strategy improving multiple traits simultaneously. This comparison is of interest to breeders because applied programs often focus almost entirely on yield (Eagles and Frey 1974; Bernardo 2010).

As a second benchmark, we used independent culling levels (ICL), i.e., only individuals meeting minimum requirements for each trait are selected (*cf*. Bernardo 2010). When ICL selection is applied to two traits in a single stage, selection gain for each trait can be estimated as ${\Delta G}_{k}=\frac{1 }{\alpha }\left({\varrho }_{{p}_{1},{y}_{k}}{Z}_{1}{I}_{2}+{\varrho }_{{p}_{2},{y}_{k}}{Z}_{2}{I}_{1}\right)$ (Wricke and Weber, 1986), where $\alpha$ corresponds to the total selected fraction and equals the product of the selected fractions for both traits ${\alpha }_{1}\times {\alpha }_{2}$; ${\varrho }_{{p}_{1},{y}_{k}}$ and ${\varrho }_{{p}_{2},{y}_{k}}$ are correlation coefficients between the genotypic value (*y*) of trait *k* and phenotypic values (*p*) of trait 1 and 2, respectively; ${Z}_{1}$ and ${Z}_{2}$ are the ordinates of the standard normal distribution at the truncation points ${x}_{1}$ and ${x}_{2}$, respectively, referring to the standardized culling levels defined by the breeder; and ${I}_{1}$ and ${I}_{2}$ are the probabilities associated with the corresponding truncation points ${x}_{1}$ and ${x}_{2}$ in a multivariate normal cumulative distribution function. The truncation points ${x}_{1}$ and ${x}_{2}$ are interdependent when a phenotypic correlation ${\varrho }_{{p}_{1},{p}_{2}}$ between traits 1 and 2 exists. The computation of the covariances between traits to obtain the required correlations ${\varrho }_{{p}_{1},{y}_{k}}$ and ${\varrho }_{{p}_{2},{y}_{k}}$ ${\varrho }_{{p}_{1},{p}_{2}}$ is presented in detail in the appendix. An extension of this formula given by Tallis (1961) to estimate selection gain for any number of correlated traits was implemented in the package *R selectiongain* (Mi et al. 2015). Finally, we computed the maximum selection gain for the ICL net merit as $\sum_{k=1}^{n}{a}_{k}{\Delta G}_{k}$ where ${a}_{k}$ is the economic weight of trait *k*. Following the approach of Hazel and Lush (1942), *ΔG*_*a*_ of ICL was determined under the optimum allocation of resources for the SH index regarding ${\alpha }_{j}, {N}_{j}, {L}_{j}, {T}_{j}$ at the stage *j.* To provide a vast range of culling levels, we tested 1000 different combinations of ${\alpha }_{1}$ and ${\alpha }_{2}$ with the only constraint of ${\alpha }_{j}={\alpha }_{1} \times {\alpha }_{2}$. After, the combination of ${\alpha }_{1}$ and ${\alpha }_{2}$ that gave the highest expected selection gain for the net merit was considered as the best allocation for stage *j* and used in this manuscript. Since the SH and base indices consistently outperformed ICL (Hazel and Lush 1942; Young 1961; Elgin et al. 1970; Vinson 1971), we implemented ICL only in few scenarios of the theoretical section and in the practical section.

## Results

**Fig. 1:**
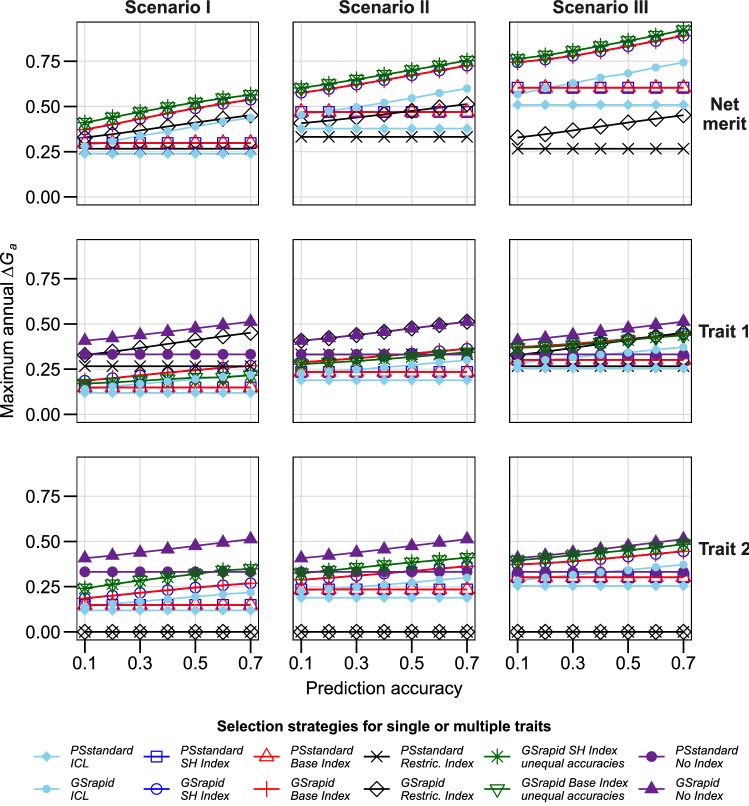
Maximum annual selection gain (*ΔG*_*a*_) for net merit and corresponding selection gain in individual traits for three selection indices in two hybrid breeding schemes. Scenarios I, II, and III with genetic correlation between traits of − 0.5, 0, and 0.5, respectively. Seven genomic prediction accuracies were evaluated. Prediction accuracies for both traits were assumed to be equal in all strategies except for “unequal accuracies”, where the accuracy of trait 1 is shown in the x axis and the accuracy of trait 2 is 0.2 higher than for trait 1 in absolute terms. For illustration, the maximum annual selection was also shown when directly selecting only one trait (“No Index”). For further details, see Table 1 and Material and methods. ICL, independent culling levels; SH, Smith–Hazel; Restric, Restricted

**Fig. 2:**
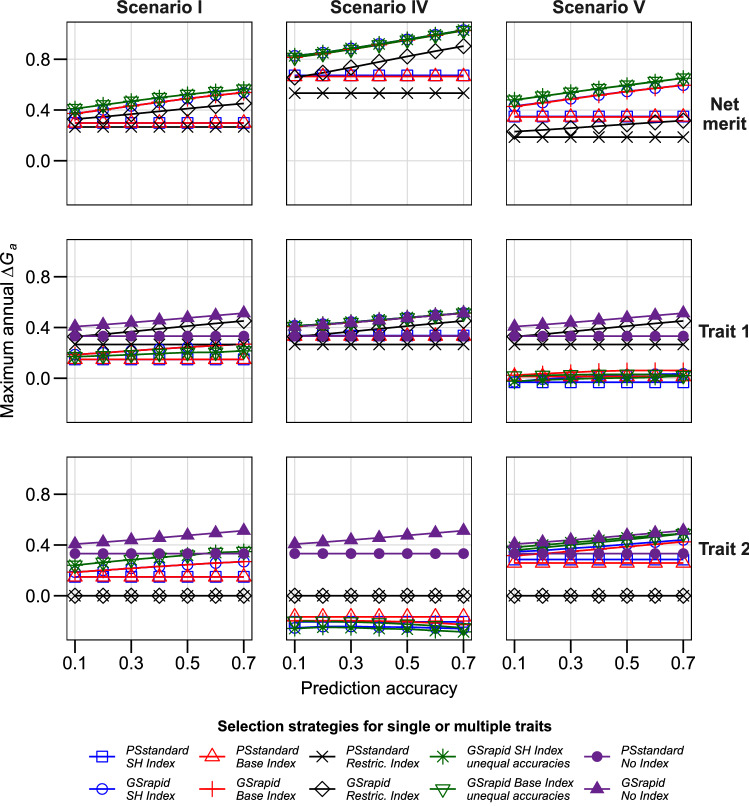
Maximum annual selection gain (*ΔG*_*a*_*)* for net merit and corresponding selection gain in individual traits for three selection indices in two hybrid breeding schemes. Scenarios I, IV, and V with economic weights of trait 1 = trait 2 = 1, trait 1 = 2 and trait 2 = 0, and trait 1 = 0.7 and trait 2 = 1.3, respectively. Seven genomic prediction accuracies were evaluated. Prediction accuracies for both traits were assumed to be equal in all strategies except for “unequal accuracies”, where the accuracy of trait 1 is shown in the x axis and the accuracy of trait 2 is 0.2 higher than for trait 1 in absolute terms. For illustration, the maximum annual selection was also shown when directly selecting only one trait (“No Index”). For further details, see Table 1 and Material and methods. SH, Smith–Hazel; Restric, Restricted

**Fig. 3:**
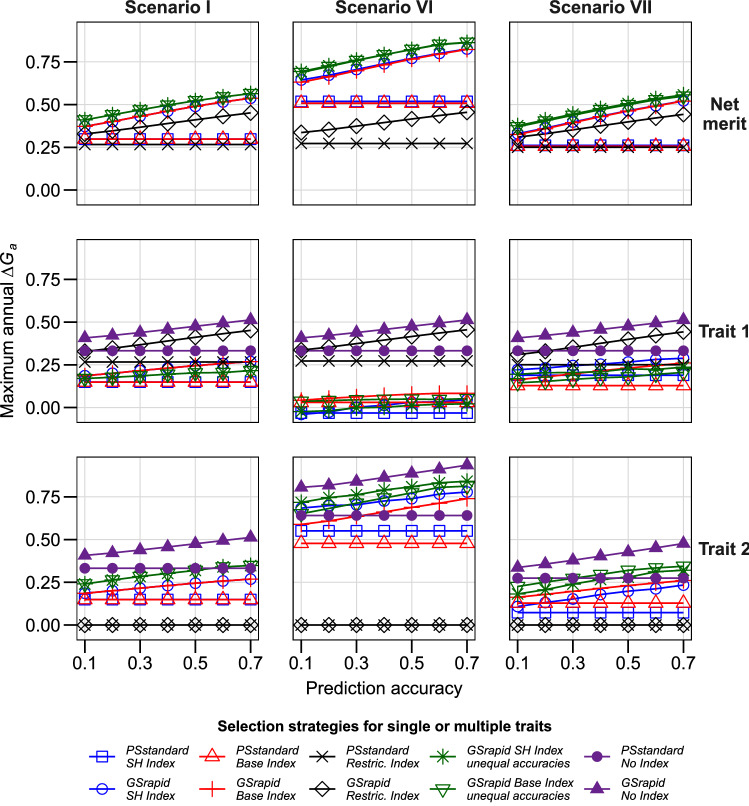
Maximum annual selection gain (*ΔG*_*a*_) for net merit and corresponding selection gain in individual traits for three selection indices in two hybrid breeding schemes. Scenarios I, VI, and VII with equal variance components between traits, three times more genetic variance for trait 2, and three times more masking variances for trait 2, respectively. Seven genomic prediction accuracies were evaluated. Prediction accuracies for both traits were assumed to be equal in all strategies except for “unequal accuracies”, where the accuracy of trait 1 is shown in the x axis and the accuracy of trait 2 is 0.2 higher than for trait 1 in absolute terms. For illustration, the maximum annual selection was also shown when directly selecting only one trait (“No Index”). For further details, see Table 1 and Material and methods. SH, Smith–Hazel; Restric, Restricted

**Fig. 4:**
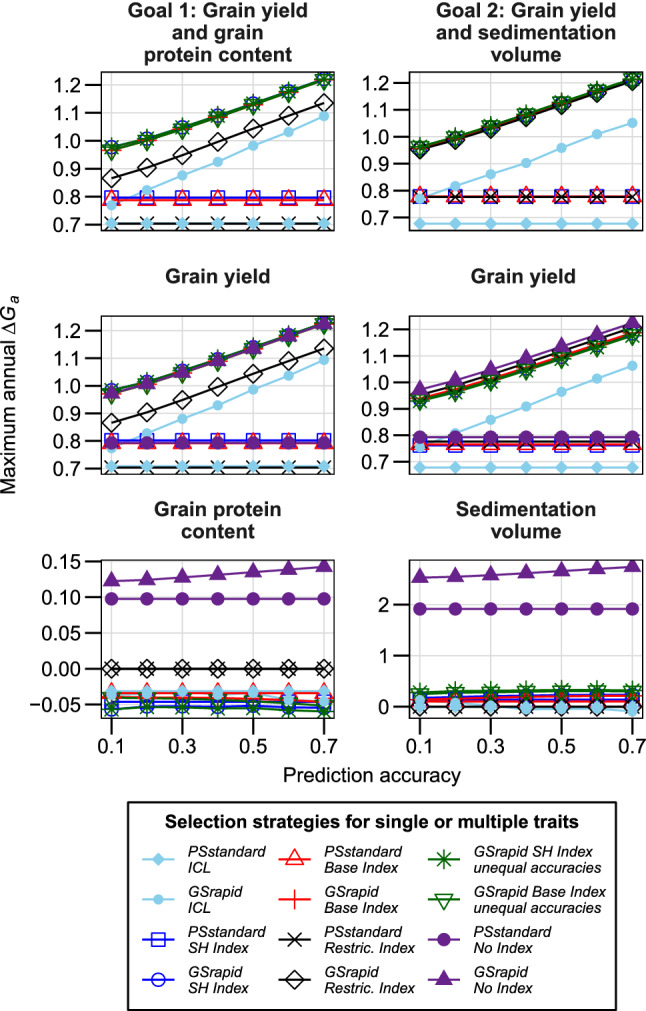
Practical section on hybrid wheat: Maximum annual selection gain (*ΔG*_*a*_*)* for net merit and corresponding selection gain in individual traits for three selection indices in two hybrid breeding schemes. Trait one was grain yield and trait two was the quality trait protein content or sedimentation volume. Variance components, the correlation between traits, and economic weights were based on empirical data as described in Goals 1 and 2 of Suppl. Table 1. Prediction accuracies for both traits were assumed to be equal in all strategies except for “unequal accuracies”, where the accuracy of trait 1 is shown in the x axis and the accuracy of trait 2 is 0.2 higher than for trait 1 in absolute terms. For illustration, the maximum annual selection was also shown when directly selecting only one trait (“No Index”). ICL independent culling levels; SH, Smith–Hazel; Restric, Restricted

For the standard scenario, *ΔG*_*a*_ for net merit was higher for the breeding strategy *GSrapid* than for *PSstandard* for all studied indices and ICL (Fig. 1, Scenario I). The three investigated selection indices offered larger *ΔG*_*a*_ for net merit in comparison to ICL (Fig. 1, Scenario I). While *ΔG*_*a*_ for net merit was almost identical for the SH and base indices for all investigated scenarios, it was lower for the restricted index (Figs. 1, 2, 3, 4, Table 2). The latter was more pronounced for breeding strategy *GSrapid* than for breeding strategy *PSstandard*. Similarly, the advantage in *ΔG*_*a*_ of *GSrapid* over *PSstandard* increased with increasing prediction accuracy for genomic selection. Assuming a higher prediction accuracy for trait 2 than for trait 1 maximized the annual selection for net merit irrespective of the scenario considered (Figs. 1, 2, 3).

**Table 2:** Practical section on hybrid wheat: Optimum allocation of test resources maximizing annual selection gain (*ΔG*_*a*_) under two alternative breeding strategies using three selection indices for the simultaneous improvement of two traits

Breeding strategy	Index	Optimum allocation of test resources								*∆G*_*a*_* Net merit*	*∆G*_*a*_* Trait 1*	*∆G*_*a*_* Trait 2*
		*N*_*ini*_	*N*_*1*_	*N*_*2*_	*N*_*3*_	*L*_*2*_	*L*_*3*_	*T*_*2*_	*T*_*3*_			
*Goal 1: Simultaneous improvement of grain yield (Trait 1) and grain protein content (Trait 2)*												
*PSstandard*	ICL	2028		507	35	5	10	1	6	0.70	0.71	− 0.03
*PSstandard*	SH	2028	−	507	35	5	10	1	6	0.80	0.80	− 0.05
*PSstandard*	Base	1892	−	473	35	6	10	1	6	0.79	0.79	− 0.03
*PSstandard*	Restricted	1892	−	473	35	6	10	1	6	0.70	0.70	0.00
*PSstandard*	No Index	1892	−	473	35	6	10	1	6	−	0.79	0.09
*GSrapid*	ICL	2652	663	124.4	−	10	−	3	−	0.88	0.88	− 0.03
*GSrapid*	SH	2652	663	124.4	−	10	−	3	−	1.05	1.05	− 0.06
*GSrapid*	Base	2652	663	124.4	−	10	−	3	−	1.04	1.05	− 0.04
*GSrapid*	Restricted	2612	653	94.5	−	10	−	4	−	0.95	0.95	0.00
*GSrapid*	No Index	2644	661	124.7	−	10	−	3	−	−	1.05	0.13
*Goal 2: Simultaneous improvement of grain yield (Trait 1) and sedimentation volume (Trait 2)*												
*PSstandard*	ICL	1892	−	473	35	6	10	1	6	0,68	0,68	0,00
*PSstandard*	SH	1892	−	473	35	6	10	1	6	0.78	0.76	0.13
*PSstandard*	Base	1892	−	473	35	6	10	1	6	0.78	0.76	0.10
*PSstandard*	Restricted	1892	−	473	35	6	10	1	6	0.78	0.78	0.00
*PSstandard*	No Index	1892	−	473	35	6	10	1	6	−	0.79	1.92
*GSrapid*	ICL	2652	663	124.4	−	10	−	3	−	0,86	0,86	0,02
*GSrapid*	SH	2652	663	124.4	−	10	−	3	−	1.03	1.01	0.21
*GSrapid*	Base	2652	663	124.4	−	10	−	3	−	1.03	1.01	0.17
*GSrapid*	Restricted	2652	663	124.4	−	10	−	3	−	1.03	1.03	0.00
*GSrapid*	No Index	2644	661	124.7	−	10	−	3	−	−	1.05	2.58

The allocation and *ΔG*_*a*_ correspond to the assumption of GS prediction accuracies of 0.3 for both traits. (ICL, independent culling levels; SH, Smith–Hazel; *N*_*ini,*_ number of lines entering the nursery assuming a selected fraction of ${\alpha }_{ini}$ = 0.25 after nursery assessment; *N*_*j*_*, L*_*j*_*, T*_*j*_ number of DH lines, test locations and testers in stages one, two, and three, respectively; No Index, maximum annual selection gain when optimization is based on trait 1 or trait 2 exclusively; only for “No Index”, *ΔG*_*a*_ for trait 1 and 2 is the maximum annual selection gain while for the other indices it is the annual selection gain for the individual traits when selected with the respective index; for further details see Suppl. Table 1)

First, we investigated the influence of the correlation of the traits, their economic weights, and variance components on *ΔG*_*a*_ in the breeding strategies and selection indices. The correlation between both traits strongly influenced *ΔG*_*a*_ and the superiority of *GSrapid* over *PSstandard* (Fig. 1)*.* For instance, when using an SH index and assuming a prediction accuracy of 0.3 for both traits, *ΔG*_*a*_ for net merit was 29.2% higher for *GSrapid* than for *PSstandard* for a positive correlation between traits of 0.5 (Fig. 1, Scenario III). This difference increased up to 44.9% for a negative correlation of -0.5 between both traits (Fig. 1, Scenario I). *ΔG*_*a*_ for net merit based on the SH or base index was considerably higher for positively than for negatively correlated traits. In contrast, for the restricted index, the maximum *ΔG*_*a*_ for net merit was obtained when traits were uncorrelated (Fig. 1, Scenario II). *ΔG*_*a*_ for net merit was always lowest for ICL except for the restricted index when traits have zero or positive correlation (Fig. 1, Scenario II and III). For all scenarios, selecting candidates based on an index resulted in selection gains for the individual traits up to 50.8% lower compared to the benchmark of selecting only for one trait (Figs. 1, 2, 3). The use of a restricted index minimized this disadvantage across almost all investigated scenarios at the expense of no selection gain in trait 2 (Figs. 1–3). The higher the correlation between the two traits, the lower was the reduction of selection gain for the individual traits when using index selection.

Second, we studied the impact of the economic weights for individual traits on *ΔG*_*a*_ net merit. Assuming a negative correlation of − 0.5 between both traits, *ΔG*_*a*_ for net merit was considerably higher for an economic weight of zero for trait 2 than for equal economic weights for both traits (Fig. 2, Scenario IV). Thereby, the selection gain expected by index selection for the individual trait 1 was comparable to that expected by selecting only for trait 1, but at the expense of a negative selection gain expected for trait 2. Assuming a two-fold higher economic weight for trait 2 than trait 1 as compared to equal weights led to a similar *ΔG*_*a*_ for net merit using the SH or base index in breeding strategy *GSrapid*. However, the selection gain expected for trait 2 was considerably higher at the expense of a reduced expected selection gain for trait 1 as compared with the standard scenario with equal weights for both traits (Fig. 2, Scenario V).

Third, we evaluated the impact of the variance components on multiple-trait selection. The higher the genetic variance or the lower the masking variance components, the higher was *ΔG*_*a*_. Even though negatively correlated to trait 1, a higher genetic variance of trait 2 led also to a higher *ΔG*_*a*_ in net merit by considerably increasing the expected selection gain for trait 2 (Fig. 3, Scenario VI). In contrast, higher non-genetic variance components in the negatively correlated trait 2 slightly reduced *ΔG*_*a*_ for net merit as well as decreased selection gain for trait 2 (Fig. 3, Scenario VII).

Calculation of an SH index requires estimates of genetic covariances between the traits. While the genetic covariance between traits is often available in breeding programs, knowledge about the covariances between genotype-by-location interactions of both traits or in hybrid breeding the covariance between SCA effects of both traits is mostly not available. Therefore, we calculated the SH index in the standard scenario also for a wide range of these covariance components and found only a small impact on *ΔG*_*a*_ for net merit and the selection gain for individual traits as well as the ranking of the different indices and breeding schemes (data not shown). Consequently, if breeders want to apply selection indices but lack the required covariance components, as a first approximation, they may use the genetic covariance between traits only and ignore the covariances between the genotype-by-location interactions of both traits and the covariance between SCA effects of both traits.

## Discussion

Plant breeders usually consider several traits during selection and apply different methods to deal with this in practice. We compared the effects of three different indices on *ΔG*_*a*_ for net merit and selection gain expected for individual traits assuming two breeding strategies: (i) classical two-stage phenotypic selection and (ii) a combination of genomic selection with one-stage phenotypic selection. In particular, we investigated the impact of variance components, correlation of traits and their economic weights on *ΔG*_*a,*_ and the ranking of the breeding strategies by varying only one factor at a time (Figs. 1, 2, 3). Finally, we demonstrate our findings in the practical section using hybrid wheat as a case of study (Fig. 4, Table 2).

### Breeding strategy *GSrapid* maximizes annual selection gain

Across all investigated scenarios, the breeding strategy *GSrapid* achieved consistently higher *ΔG*_*a*_ than *PSstandard* (Figs. 1, 2, 3, 4, Table 2). Already visible for low prediction accuracies of genomic selection, this advantage increased considerably with increasing prediction accuracies for both traits. Prediction accuracies for genomic selection across different breeding cycles were reported to be fairly low (cf.Zhao et al. 2013; Albrecht et al. 2014; Michel et al. 2016; Rapp et al. 2018). However, assuming a prediction accuracy for genomic selection for both traits of only 0.3 in the standard scenario and taking the SH index led already to 44.9% *ΔG*_*a*_ for *GSrapid* compared with *PSstandard* (Fig. 1, Scenario I). This advantage depends on the correlation of traits, economic weights, and variance components of the individual traits. For instance, the advantage decreases to 35.9% when a larger genetic variance was assumed for the second trait (Fig. 3, Scenario VI). The consistent advantage of the breeding strategy *GSrapid* over *PSstandard* corroborates a previous study, which investigated single-trait improvement (grain yield) across several crops (Marulanda et al. 2016). As already emphasized by these authors, the advantage of breeding strategy *GSrapid* is based on two pillars: the shorter breeding cycle length and the higher number of DH lines initially tested (Table 2). Here, we confirmed that using genomic selection in breeding programs improving two traits simultaneously is promising regardless of the prediction accuracy of the individual traits. Even if prediction accuracy is 0, *GSrapid* provided a more considerable expected genetic gain for the net merit than *PSstandard.* This is because it can be extrapolated from the linear trajectories of the expected genetic gain when prediction accuracy increases from 0.1 to 0.7 in Figs. 1, 2, 3, 4. Assuming a higher prediction accuracy for trait 2 than for trait 1, *ΔG*_*a*_ for net merit was increased for the use of an SH or base index even under the unfavorable situation that trait 2 was negatively correlated with trait 1 (Fig. 1, Scenario I). In conclusion, the integration of genomic selection into applied breeding programs appears very promising, but the combination of traits requires special attention for choosing the selection index. If not explicitly stated otherwise, we concentrate the following discussion on breeding strategy *GSrapid*.

### Choice of the index largely influences selection gain expected for individual traits

The choice of the index had a large impact on *ΔG*_*a*_ for net merit (Figs. 1, 2, 3). Almost identical *ΔG*_*a*_ for net merit were determined for the use of SH or base index across all considered scenarios. By contrast, *ΔG*_*a*_ for net merit using a restricted index was considerably lower for both breeding strategies *GSrapid* and *PSstandard* depending on the investigated scenario. For instance, the superiority in *ΔG*_*a*_ for net merit using SH or base index compared to restricted index increased from 13.5% for negatively correlated traits (Fig. 1, Scenario I) to 112.2% for positively correlated traits (Fig. 1, Scenario III). This is explained by the efficiency of SH and base indices to exploit the positive correlation between traits by selecting the best genotypes for both traits to maximize the expected genetic gain.

On the other hand, the restricted index suffered when applied in positively or negatively correlated traits as the best genotypes for the trait improvement can no longer be selected by the breeders because they do not fit the goal of the index. Similar reductions in the realized genetic gain when applying restricted index for positively correlated traits in oats were found by Rosielle and Frey (1975). Among the available restricted indices, Kempthorne and Nordskog (1959) proposed to set the most restrictive conditions to maximize the gain of one trait while keeping the second at the desired level. Future use of restricted indices in the *selectiongain* package might include more versatile alternatives such as those in Pesek and Baker (1969).

The choice of the index not only influenced the *ΔG*_*a*_ for net merit, but also the expected selection gain for the individual traits combined in the index (Figs. 1, 2, 3). For instance, for the standard scenario and prediction accuracies between 0.1 and 0.7, the selection gains expected for trait 1 applying SH or base index were 54.3·% and 47.7% lower than the *ΔG*_*a*_ of strategies improving only one trait. This negative effect on realized selection gain in individual traits was especially pronounced when traits were negatively correlated, underpinning that parallel selection on two traits is always a compromise regarding the selection gain for each of the traits. Similarly, the more weight is given to one trait in the index, the lower is the selection gain for the other trait. By contrast, the use of a restricted index decreased the disadvantage of reduced individual selection gain for trait 1 in index selection across almost all investigated scenarios but at the expense of no selection gain for trait 2.

While the correlation between target traits can hardly be changed and variance components only with considerable expenditures, breeders can easily influence the choice of the economic weights and indices. Economic weights should reflect market demands and numerous methods are at disposal, a topic beyond the scope of this study (cf. Graffius 1964; Eagles and Frey 1974; Mistele et al. 1994; Bernardo 2010). As expected, the higher the weight given to an individual trait, the larger is its individual selection gain by applying index selection (Fig. 2). However, the correlation between traits largely affected the selection gain for the other trait in index selection. While for positively correlated traits an indirect selection gain can be expected in index selection, in the case of negatively correlated traits each slight increase of the economic weight of trait 1 will automatically reduce the selection gain for trait 2 and vice versa (Fig. 2, Scenario IV and V).

The major difference between restricted and SH index or base index is in the specific economic significance assigned to the traits to warrant the desired selection gain for each of them. Using an SH or base index aims at maximizing the selection gain (*ΔG*_*a*_) for net merit by ideally increasing also the selection gain for each trait. However, for negatively correlated traits and depending on the variance components and economic weights of the individual traits, the use of an SH or base index can lead to a negative selection gain for an individual trait (Fig. 2, Scenario IV). By contrast, using a restricted index implicitly includes the decision that trait 2 is less important than trait 1. Independent of the trait correlations and variance components, this warrants a non-negative selection gain for trait 2 and therefore represents an interesting option for negatively correlated traits, as long as the market requirement for trait 2 is satisfied by keeping it just constant.

Choosing either an SH or a base index yielded similar results for *ΔG*_*a*_ on net merit across almost all scenarios. For an SH index, however, information about several covariance components is required, which are mostly not available in applied breeding programs. Furthermore, the base index seems slightly more robust in the selection gain for individual traits than the SH index. For instance, a higher genetic variance of trait 2 led to a more intensive selection on this trait compared to the base index (Fig. 3, Scenario VI and VII, respectively). Thus, we recommend using the base index for applied breeding programs.

### Advantages of index selection over ICL in GS based strategies

In applied breeding, phenotypic data of quality-related traits is available weeks or months after agronomic data. Then, selection based on indices is delayed until all data required for the index are completed. Breeding programs facing this data production imparity, have routinely used ICL to avoid delays in selections and reduce the number of genotypes assessed for quality-related traits. However, the wide adoption of genomic selection in plant breeding favors index selection over ICL, as GEBV are simultaneously available when the calibration set uses data from previous years. We found that ICL based strategies obtained 28.1% and 23.9% lower *ΔG*_*a*_ for net merit as compared to the SH index for a GS prediction accuracy of 0.3 and a genetic correlation between traits of − 0.5 and 0.5 respectively (Fig. 1). This inferiority remained across all levels of correlation between traits or accuracy of genomic prediction, which is in harmony with the literature on phenotypic selection (Hazel and Lush 1942; Young 1961; Elgin et al 1970; and Vinson 1971). Therefore, we discourage the uncritical use of ICL in applied breeding but acknowledge specific scenarios where it might still be appropriate in the short term. For instance, when the rate of propagating material produced per plant is low, several cycles of multiplication are required before conducting yield trials. Thus, reliable information on yield is obtained one or several seasons after highly heritable traits such as plant height or yield components have been observed. In this case, ICL offers an alternative to practice multistage selection for multiple traits (Bernardo, 2010) by applying phenotypic selection on secondary traits during the first stages and in a later stage on yield. An alternative for these breeding programs facing data production imparity is to move from *PSstandard* ICL to *GSrapid* SH or base index. In this case, index selection based on GEBV during the initial stages is carried out in parallel to the planting for material multiplication. Selection based on phenotypic assessment takes place on a drastically reduced number of genotypes in a subsequent stage. Current developments in genome analysis and the low genotyping costs facilitate the application of this breeding strategy even in polyploid or “orphan” crops (Kamenya et al. 2021). Moreover, calibration set design could keep prediction accuracy and facilitate the selection of individuals to maintain the calibration set. A detailed assessment of the advantage of index selection over ICL in breeding programs with data production imparity exceeds the scope of our manuscript but will be investigated using the new implementation of ICL and index selection in the R package “*selectiongain*”*.*

### Practical section: index selection on grain yield and quality in hybrid wheat

In wheat, the combination of high grain yield with good baking quality is of utmost importance in the development of new varieties and the remuneration of farmers (Laidig et al. 2016). As baking trials are expensive and time-consuming, breeders and traders alike usually estimate bread-making quality by the grain protein content and the sedimentation volume (cf. Thorwarth et al. 2018). We, therefore, investigated two goals: the simultaneous improvement of (1) grain yield with grain protein content and (2) grain yield with sedimentation volume based on empirical data for variance components, correlation of traits, and economic weights. For both goals, selection gain for net merit and the individual traits was considerably higher for the breeding strategy *GSrapid* than for *PSstandard* (Fig. 4, Table 2). ICL was least efficient to improve the net merit for both goals, matching our theoretical results for the negative correlation between traits (Fig. 1, Scenario I). Using the SH or base index, the negative correlation of -0.40 between grain yield and grain protein content (goal 1) coupled with a low economic weight for grain protein content resulted in a negative selection gain for this trait. The use of a restricted index avoids a reduction of grain protein content but at the expense of a reduction in *ΔG*_*a*_ for net merit and grain yield. However, the restricted index gives higher weight to grain protein content than based on the marginal returns of farmers over the last years, underpinning the importance of the choice of the index and the economic weights for individual traits on short- and long-term selection gain.

Although sedimentation volume was negatively correlated (r = -0.18) with grain yield, their combination in the SH or base index resulted in positive *ΔG*_*a*_ for net merit and for each trait (Fig. 4, goal 2; Table 2). The *ΔG*_*a*_ observed for the individual traits is most likely due to the low genetic correlation of traits and the high genetic variance and heritability of sedimentation volume. Despite this positive result, aiming for goal 2 led to 2.5% less *ΔG*_*a*_ for net merit compared to goal 1. Similarly, 3.8% less selection gain for grain yield was obtained when comparing goal 2 to goal 1. Delivering almost identical *ΔG*_*a*_ for net merit and grain yield, the use of an SH index resulted in a slightly higher *ΔG*_*a*_ for sedimentation volume than the base index. Interestingly, the advantage of the SH or base index over a restricted index was low for *ΔG*_*a*_ in net merit and grain yield but more pronounced for sedimentation volume.

The optimum allocation of test resources, i.e., optimum number of test candidates, locations, and testers in the individual selection stages, was similar across the different indices for both goals (Table 2). A similar number of test candidates, however, does not necessarily mean that the same genotypes were selected by different indices. By contrast, the different selection gains in the individual traits showed that quite different genotypes were finally selected depending on the index and the investigated scenario (Figs. 1, 2, 3). This is in line with experimental findings in bread (Thorwarth et al. 2018) and durum wheat (Rapp et al. 2018). Although quite similar across the indices in a given scenario, the optimum allocation of resources was influenced by the correlation of traits, economic weights, and variance components of both traits. For breeding strategy *GSrapid* using the SH index, we found in our theoretical section the average number of initial DH lines to be (1) 16.4% higher for negatively than for positively correlated traits, (2) 12.5% higher for equal economic weights for both traits versus all economic importance devoted to one trait, and (iii) 3.6% higher for equal variance components for both traits in comparison to larger masking variance for one of the traits (data not shown). This underlines the need to optimize the allocation of test resources of breeding programs separately and routinely depending on the target traits and markets.

In conclusion, the breeding strategy combining genomic selection with one-stage phenotypic selection (*GSrapid*) had a considerably higher *ΔG*_*a*_ than the breeding strategy based on two-stage phenotypic selection (*PSstandard*) regardless of the index chosen for combining two traits. However, the choice of an index and the economic weight given to the individual traits have a large effect on *ΔG*_*a*_ for the net merit (index) as well as for the individual traits. Breeders should therefore carefully investigate which index fits best to the short- and long-term goals of their breeding program. With the extensions of the R package “*selectiongain*” developed for this dissertation, breeders are now able to rapidly determine the optimum allocation of test resources and *ΔG*_*a*_ for line and hybrid crops with different breeding strategies and choosing among index selection procedures.

### Electronic supplementary material

Below is the link to the electronic supplementary material.

Supplementary file1 (DOCX 14 kb)

Supplementary file2 (EPS 41 kb)

Supplementary file1 (R 11 kb)

## Appendix

### Variances and covariances required for estimating the expected selection gain in hybrid breeding programs for simultaneous improvement of two traits

#### (i) Optimum index for two traits in one stage of phenotypic selection

First, we define two matrices ${\varvec{G}}$ **and** ${\varvec{P}}$. ${\varvec{G}}$ refers to the genetic (co-)variances of the two traits (*k* and *d*)

1 $${\varvec{G}} = \left[ {\begin{array}{*{20}c} {\sigma_{{{\text{GCA}}_{{\text{k}}} }}^{2} } & {cov_{{{\text{GCA}}_{{\left( {\text{k,d}} \right)}} }} } \\ {cov_{{{\text{GCA}}_{{\left( {\text{k,d}} \right)}} }} } & {\sigma_{{{\text{GCA}}_{{\text{d}}} }}^{2} } \\ \end{array} } \right]$$

where ${\sigma }_{{GCA}_{k}}^{2}$ is the variance of general combining ability (GCA) effects of trait $k$, ${\sigma }_{{GCA}_{d}}^{2}$ the variance of general combining ability effects of trait $d$, and ${cov}_{{GCA}_{(k,d)}}$ the covariance of general combining ability effects between trait $k$ and $d$. These variances and covariances are estimated (e.g., using REML) from field trials representing the experimental populations of the breeding program. ${\varvec{P}}$ refers to the respective phenotypic (co)variances:

2 $$P = \left[ {\begin{array}{*{20}c} {\sigma_{{{\text{P}}_{{\text{k}}} }}^{2} } & {{\text{cov}}_{{\text{P(k,d)}}} } \\ {{\text{cov}}_{{\text{P(k,d)}}} } & {\sigma_{{{\text{P}}_{{\text{k}}} }}^{2} } \\ \end{array} } \right]$$

where ${\sigma }_{{P}_{k}}^{2}$ is the phenotypic variance of trait $k$ with:

3 $$\begin{gathered} \sigma_{{{\text{P}}_{{\text{k}}} }}^{2} = \sigma_{{{\text{GCA}}_{{\text{k}}} }}^{2} + \frac{{\sigma_{{({\text{GCA}} \times {\text{Y}})_{{\text{k}}} }}^{2} }}{Y} + \frac{{\sigma_{{\left( {{\text{GCA}} \times {\text{L}}} \right)_{{\text{k}}} }}^{2} }}{L} \hfill \\ \;\;\;\;\;\;\;\; + \frac{{\sigma_{{\left( {{\text{GCA}} \times {\text{Y}} \times {\text{L}}} \right)_{{\text{k}}} }}^{2} }}{Y \times L} + \frac{{\sigma_{{{\text{SCA}}_{{\text{k}}} }}^{2} }}{T} + \frac{{\sigma_{{({\text{SCA}} \times {\text{Y}})_{{\text{k}}} }}^{2} }}{T \times Y} \hfill \\ \;\;\;\;\;\;\;\; + \frac{{\sigma_{{\left( {{\text{SCA}} \times {\text{L}}} \right)_{{\text{k}}} }}^{2} }}{T \times L} + \frac{{\sigma_{{\left( {{\text{SCA}} \times {\text{Y}} \times {\text{L}}} \right)_{{\text{k}}} }}^{2} }}{T \times Y \times L} + \frac{{\sigma_{{{\text{e}}_{{\text{k}}} }}^{2} }}{T \times Y \times L \times R}, \hfill \\ \end{gathered}$$

where ${\sigma }_{({GCA\times Y)}_{k}}^{2}$ is the variance of GCA $\times$ Year interactions for trait $k$, ${\sigma }_{({GCA\times L)}_{k}}^{2}$ the variance of GCA $\times$ Location interactions for trait $k$, ${\sigma }_{({GCA\times Y\times L)}_{k}}^{2}$ the variance of GCA $\times$ Year $\times$ Location interactions for trait $k$, ${\sigma }_{{SCA}_{k}}^{2}$ the variance of specific combining ability effects (SCA) for trait $k, {\sigma }_{({SCA\times Y)}_{k}}^{2}, {\sigma }_{{(SCA\times L)}_{k}}^{2},{\sigma }_{{(SCA\times Y\times L)}_{k}}^{2}$ the respective interactions with SCA and ${\sigma }_{{e}_{k}}^{2}$ the variance of plot error for trait $k$. $T, Y,L,$ and $R$ refer to the number of testers, years, locations, and replicates used in the field trials. $Y$ is in most cases equal to 1 as selections after phenotypic evaluation are generally conducted after one year of testing. The phenotypic variance for trait $d$ (${\sigma }_{{P}_{d}}^{2}$) is estimated similarly as ${\sigma }_{{P}_{k}}^{2}$. Finally, the phenotypic covariance between traits $k$ and *d* can be expressed as:

4 $$\begin{gathered} {\text{cov}}_{p(k,d)} = {\text{cov}}_{GCA(k,d)} + \frac{{{\text{cov}}_{GCA \times Y(k,d)} }}{Y} + \frac{{{\text{cov}}_{GCA \times L(k,d)} }}{L} \hfill \\ \;\;\;\;\;\;\;\;\;\;\;\;\;\;\;\frac{{{\text{cov}}_{GCA \times Y \times L(k,d)} }}{Y \times L} + \frac{{{\text{cov}}_{SCA(k,d)} }}{T} + \frac{{{\text{cov}}_{SCA \times Y(k,d)} }}{T \times Y} \hfill \\ \;\;\;\;\;\;\;\;\;\;\;\;\;\;\;\frac{{{\text{cov}}_{SCA \times L(k,d)} }}{T \times L} + \frac{{{\text{cov}}_{SCA \times Y \times L(k,d)} }}{T \times Y \times L} + \frac{{{\text{cov}}_{e(k,d)} }}{T \times Y \times L \times R} \hfill \\ \end{gathered}$$

where the covariances on the right-hand side are defined as the variances on the right-hand side of Eq. (3) except that they refer to the two traits $k$ and $d$ measured in the same locations and years.

According to the formulation of the optimum index of Smith (1936), the matrices ${\varvec{G}}$ and ${\varvec{P}}$ and the vector of economic weights ${\varvec{a}}$ can be used to determine the vector of phenotypic weights ${\varvec{b}}$ (Wricke and Weber 1986):

5 $${\varvec{b}} = \user2{ P}^{ - 1} {\varvec{Ga}}$$

For two traits ($k$ and $d$), we have $b' = \left[ {b_{k} ,b_{d} } \right]$ and commonly the phenotypic weights are expressed as relative to the first element of the vector, i.e., $b' = \left[ {1,~\frac{{b_{d} }}{{b_{k} }}} \right]$ (Wricke and Weber 1986). If the vector ${\varvec{b}}$ is obtained with Eq. (5), the variance $\sigma_{I}^{2}$ of the phenotypic index $\left( I \right)$ equals covariance of $I$ with the net merit $\left( H \right)$ $cov_{{\left( {I,H} \right)}}$ (Wricke and Weber 1986):

6 $$\sigma_{{\text{I}}}^{2} = b^{\prime}Pb = b^{\prime}Ga = {\text{cov}}_{{\left( {\text{I,H}} \right)}}$$

#### (ii) Optimum index in two-stage phenotypic selection

We propose to compute the index for the net merit $H$, and two phenotypic indices $I_{1}$ and $I_{2}$ corresponding to each stage of phenotypic assessment. Thus, five covariances are required for computation of the selection gain: $\sigma_{{I_{1} }}^{2}$, $\sigma_{{I_{2} }}^{2}$, $cov_{{\left( {I_{1} ,H} \right)}} , { }cov_{{\left( {I_{2} ,H} \right)}}$ and $cov_{{\left( {I_{1} ,I_{2} ,} \right)}}$. The genetic matrices ${\varvec{G}}$ are defined as:

7 $$G_{1} = G_{2} = \left[ {\begin{array}{*{20}c} {\sigma_{{{\text{GCA}}_{{\text{k}}} }}^{2} } & {{\text{cov}}_{{{\text{GCA}}_{{\left( {\text{k,d}} \right)}} }} } \\ {{\text{cov}}_{{{\text{GCA}}_{{\left( {\text{k,d}} \right)}} }} } & {\sigma_{{{\text{GCA}}_{{\text{d}}} }}^{2} } \\ \end{array} } \right]$$

where ${\varvec{G}}_{1}$ corresponds to stage 1 and ${\varvec{G}}_{2}$ to stage 2. As selection is performed in both traits in both stages, ${\varvec{G}}_{1} = \user2{ G}_{2}$**.** The phenotypic matrices ${\varvec{P}}$ are likewise defined with corresponding meanings:

8 $$P_{1} = \left[ {\begin{array}{*{20}c} {\sigma_{{{\text{P1}}_{{\text{k}}} }}^{2} } & {{\text{cov}}_{{\text{P1(k,d)}}} } \\ {{\text{cov}}_{{\text{P1(k,d)}}} } & {\sigma_{{{\text{P1}}_{{\text{k}}} }}^{{2}} } \\ \end{array} } \right]\;and\;P_{2} = \left[ {\begin{array}{*{20}c} {\sigma_{{{\text{P2}}_{{\text{k}}} }}^{{2}} } & {{\text{cov}}_{{\text{P2(k,d)}}} } \\ {{\text{cov}}_{{\text{P2(k,d)}}} } & {\sigma_{{{\text{P2}}_{{\text{k}}} }}^{{2}} } \\ \end{array} } \right]$$

The definition and notations of the variances and covariances can be directly extrapolated from Eq. (2).

After obtaining the matrices ${\varvec{G}}_{1}$, ${\varvec{G}}_{2}$, ${\varvec{P}}_{1}$ and ${\varvec{P}}_{2}$, we can calculate the phenotypic weights for the optimum index:

9 $${\varvec{b}}_{1} = P_{1}^{ - 1} G_{1} a \;and\; {\varvec{b}}_{2} = P_{2}^{ - 1} G_{2} a$$

and after standardized according to the first element, we get:

10 $$b_{1}^{^{\prime}} = \left[ {1,\frac{{b_{1d} }}{{b_{1k} }}} \right]\;and\;b_{2}^{^{\prime}} = \left[ {1,\frac{{b_{2d} }}{{b_{2k} }}} \right]$$

The variances of the phenotypic indices in stages 1 and 2 ($\sigma_{{I_{1} }}^{2}$ and $\sigma_{{I_{2} }}^{2}$) and the covariances between the net merit and the phenotypic indices in stages 1 and 2 ($cov_{{\left( {I_{1} ,H} \right)}}$ and $cov_{{\left( {I_{2} ,H} \right)}}$) are obtained as:

11 $${\varvec{\sigma}}_{{{\varvec{I}}_{1} }}^{2} = b_{1}^{^{\prime}} P_{1} b_{1 }\; and\; {\varvec{\sigma}}_{{{\varvec{I}}_{2} }}^{2} = b_{2}^{^{\prime}} P_{2} b_{2}$$

12 $${\text{cov}}_{{\left( {I_{1} ,H} \right)}} = b_{1}^{^{\prime}} {\text{Ga}} \;{\text{and}} \;{\text{cov}}_{{\left( {I_{2} ,H} \right)}} = b_{2} ^{\prime}{\text{Ga}}$$

Finally, to estimate the covariance between the phenotypic indices in the two stages $cov_{{\left( {I_{1} ,I_{2} } \right)}}$, the phenotypic covariance for trait $k$ in different stages is estimated as follows: (Utz 1969),

13 $${\text{cov}}_{{p(k_{1} ,d_{2} )}} = \sigma_{{GCA_{k} }}^{2} + \frac{{\sigma_{{\left( {GCA \times L} \right)_{k} }}^{2} }}{{L_{c} }}$$

where $L_{c}$ is the number of common locations between stages 1 and 2. For trait $d$, the covariance between the phenotypic indices in two stages ${\text{cov}}_{{p(d_{1} ,d_{2} )}}$ is estimated correspondingly. The phenotypic covariance between trait $k$ in stage 1 and trait $d$ in stage 2 is defined as (Utz 2016, personal comm):

14 $${\text{cov}}_{{P(k_{1} ,d_{2} )}} = {\text{cov}}_{GCA(k,d)} + \frac{{{\text{cov}}_{GCA \times L(k,d)} }}{{L_{c} }}$$

which also corresponds to the phenotypic covariance between trait $d$ in stage 1 and trait $k$ in stage 2. The above-mentioned variances were organized in matrix ${\varvec{W}}$

15 $$W = \left[ {\begin{array}{*{20}c} {{\text{cov}}_{{{\text{p(k}}_{{1}} {\text{,k}}_{{2}} {)}}} } & {{\text{cov}}_{{{\text{p(k}}_{{1}} {\text{,d}}_{{2}} {)}}} } \\ {{\text{cov}}_{{{\text{p(d}}_{{1}} {\text{,k}}_{{2}} {)}}} } & {{\text{cov}}_{{{\text{p(d}}_{{1}} {\text{,d}}_{{2}} {)}}} } \\ \end{array} } \right]$$

Finally, the covariance between the phenotypic indices in the two stages is (Utz 2016, personal communication):

16 $$cov_{{\left( {I_{1} ,I_{2} } \right)}} = {\varvec{b}}_{1} ^{\prime}{\varvec{Wb}}_{2}$$

#### (iii) Optimum index for one stage of genomic selection

Following Dekkers (2007), we present the matrix of genetic variances for the two traits based on genomic estimated breeding values (${\varvec{G}}_{{{\varvec{MEBV}}}}$) as follows

17 $${\varvec{G}}_{{{\text{MEBV}}}} = \left[ {\begin{array}{*{20}c} {\sigma_{{{\text{MEBV}}_{{\text{k}}} }}^{{2}} } & {cov_{{{\text{MEBV}}_{{\left( {\text{k,d}} \right)}} }} } \\ {cov_{{{\text{MEBV}}_{{\left( {\text{k,d}} \right)}} }} } & {\sigma_{{{\text{MEBV}}_{{\text{d}}} }}^{{2}} } \\ \end{array} } \right]$$

where $\sigma_{{MEBV_{k} }}^{2}$ is the variance of molecular estimated breeding values (MEBV) for the trait $k$, $\sigma_{{MEBV_{d} }}^{2}$ is the variance of MEBV for the trait $d$ and $cov_{{MEBV_{{\left( {k,d} \right)}} }}$ is the covariance of molecular estimated breeding values for traits $k$ and $d$. The variances of molecular estimated breeding values for one trait (e.g., trait $k$) can be estimated as (Dekkers 2007):

18 $$\sigma_{{{\text{MEBV}}_{{\text{k}}} }}^{{2}} = \left( {r_{{{\text{MG}}_{{\text{k}}} }} \times \sigma_{{{\text{G}}_{{\text{k}}} }} } \right)^{2}$$

where $r_{{MG_{k} }}$ is the correlation of the MEBV and the total genetic value and represents the accuracy of MEBV in genomic selection (Meuwissen et al. 2001), $\sigma_{{G_{k} }}$ is the genetic standard deviation of trait $k$, which in the case of hybrids is $\sigma_{{GCA_{k} }}$. The covariance of MEBV between traits $k$ and $d$ $cov_{{MEBV_{{\left( {k,d} \right)}} }}$ is obtained from correlation formulas presented by Dekkers (2007):

19 $${\varvec{corr}}_{{{\varvec{MEBV}}_{{\left( {{\varvec{k}},{\varvec{d}}} \right)}} }} = \frac{{cov_{{MEBV_{{\left( {k,d} \right)}} }} }}{{\sqrt { \sigma_{{MEBV_{k} }}^{2} \times \sigma_{{MEBV_{d} }}^{2} } }}$$

And thus,

$${\text{cov}}_{{MEBV_{(k,d)} }} = {\text{cov}}_{{MEBV_{(k,d)} }} \times \sigma_{{MEBV_{k} }} \times \sigma_{MEBVd}$$

and replacing $corr_{{MEBV_{{\left( {k,d} \right)}} }}$ by $r_{{\hat{Q}_{k} }} \times r_{{\hat{Q}_{d} }} \times \frac{{cov_{{GCA_{{\left( {k,d} \right)}} }} }}{{\sqrt { \sigma_{{GCA_{k} }}^{2} \times \sigma_{{GCA_{d} }}^{2} } }}$ (Dekkers 2007), we get:

$${\varvec{cov}}_{{{\varvec{MEBV}}_{{\left( {{\varvec{k}},{\varvec{d}}} \right)}} }} = r_{{\hat{Q}_{k} }} \times r_{{\hat{Q}_{d} }} \times \frac{{cov_{{GCA_{{\left( {k,d} \right)}} }} }}{{\sqrt { \sigma_{{GCA_{k} }}^{2} \times \sigma_{{GCA_{k} }}^{2} } }} \times \sigma_{{MEBV_{k} }} \times \sigma_{{MEBV_{d} }}$$

where $r_{{\hat{Q}_{k} }}$ and $r_{{\hat{Q}_{d} }}$ are the accuracies of the MEBV as predictors of the marker-associated genetic effects for the traits $k$ and $d,$ respectively. Replacing $\sigma_{{MEBV_{k} }}$ and $\sigma_{{MEBV_{d} }}$ by the accuracy of GS and the genetic standard deviation, as described before, yields:

$${\varvec{cov}}_{{{\varvec{MEBV}}_{{\left( {{\varvec{k}},{\varvec{d}}} \right)}} }} =\; r_{{\hat{Q}_{k} }} \times r_{{\hat{Q}_{d} }} \times \frac{{cov_{{GCA_{{\left( {k,d} \right)}} }} }}{{\sqrt { \sigma_{{GCA_{k} }}^{2} \times \sigma_{{GCA_{d} }}^{2} } }} \times r_{{MG_{k} }} \times \sigma_{{GCA_{k} }} \times r_{{MG_{d} }} \times \sigma_{{GCA_{d} }}$$

Canceling terms, we finally obtain the covariance of MEBV between traits $k$ and $d$ as:

20 $${\text{cov}}_{{MEBV_{(k,d)} }} = r_{{\hat{Q}_{k} }} \times r_{{\hat{Q}_{d} }} \times r_{{MG_{k} }} \times r_{{MG_{d} }} \times {\text{cov}}_{GCA(k,d)}$$

For the computation $r_{{\hat{Q}_{k} }}$ Dekkers (2007) proposed:

21 $$r_{{\hat{Q}_{k} }} = \frac{{r_{{MG_{k} }} }}{{q_{k} }}$$

where $q_{k}^{2}$ is the proportion of genetic variance that is associated with markers. This parameter can be (i) estimated from experimental data via REML or (ii) calculated using population and genetic parameters of the crop of interest as proposed by Goddard et al. (2011). The correlation $r_{{\hat{Q}_{d} }}$ can be estimated using a similar approach.

Following Dekkers (2007), the matrices ${\varvec{G}}_{{{\varvec{MEBV}}}}$ and ${\varvec{P}}_{{{\varvec{MEBV}}}}$ are equal because the heritability of MEBV is assumed to be 1. Thus,

22 $${\varvec{b}} = P_{MEBV}^{ - 1} \times G_{MEBV} \times a = \user2{ a}$$

so that in the optimum index formulation for genomic selection, the phenotypic weights are equal to the economic weights, which agrees with the genomic selection index proposed by Ceron-Rojas et al. (2015). Consequently, for genomic selection, the optimum and the base index are identical. Expressing the phenotypic weights as relative ratios of $b_{k}$, we can proceed to compute the variance of the phenotypic index $\sigma_{I}^{2}$, and covariance of the phenotypic index with the net merit $cov_{{\left( {I,H} \right)}}$ as in Eq. (6)

#### (iv) Optimum index for one stage of genomic selection followed by one or two-stage(s) of phenotypic selection

We assume that the matrices ${\varvec{G}}$ and ${\varvec{P}}$ for both traits have been computed as shown in section (*i)* for the case of phenotypic selection and section (*iii)* for the case of genomic selection. Additionally, the variances of the phenotypic indices $\sigma_{I}^{2}$ and the covariances $cov_{{\left( {I,H} \right)}}$ between the net merit and the phenotypic indices in stages of phenotypic and genomic selection are also computed as shown in sections (*i)* and (*iii),* respectively. Here, estimation of the covariance $cov_{{\left( {I_{1} ,I_{2} } \right)}}$ between the phenotypic indices in the two selection stages is important and we provided in the following a detailed derivation.

The phenotypic covariance ${\text{cov}}_{{p(k_{1} ,k_{2} )}}$ of trait $k$ between the first stage of genomic selection (GS) and the second stage of phenotypic selection (PS) is derived from the formulas presented in Table 1 of Dekkers (2007):

23 $${\text{co}} rr_{{p_{{(k_{1} ,k_{2} )}} }} = r_{{MG_{k} }} \times h_{k}$$

where $corr_{{p(k_{1} ,k_{2} )}}$ is the correlation between GEBV and phenotypes for trait $k$ and $h_{k}^{2}$ is the heritability for trait $k$. We express the correlation and the heritability as variances and covariances:

$$\frac{{{\text{cov}}_{{p(k_{1} ,k_{2} )}} }}{{\sqrt {\sigma_{{pk_{1} }}^{2} \times \sigma_{{pk_{2} }}^{2} } }} = r_{{MG_{k} }} \times \frac{{\sigma_{{GCA_{k} }} }}{{\sigma_{{pk_{2} }} }}$$

And thus,

$${\text{cov}}_{{p(k_{1} ,k_{2} )}} = r_{{MG_{k} }} \times \frac{{\sigma_{{GCA_{k} }} }}{{\sigma_{pk2} }} \times \sigma_{pk1} \times \sigma_{pk2}$$

This expression can be simplified by canceling out the term $\sigma_{{p_{{k_{2} }} }}$ and replace $\sigma_{{p_{{k_{1} }} }}$(which is equal to $\sigma_{{MEBV_{k} }}$) by $r_{{MG_{k} }} \times \sigma_{{GCA_{k} }}$ as explained in section (*iii):*

24 $${\text{cov}}_{{p(k_{1} ,k_{2} )}} = r_{{MG_{k} }} \times \sigma_{{GCA_{k} }} \times r_{{MG_{k} }} \times \sigma_{{GCA_{k} }} = \sigma^{2}_{{GCA_{k} }} \times r^{2}_{{MG_{k} }}$$

The phenotypic covariance of trait $d$ between the first stage of genomic selection (GS) followed by the second stage of phenotypic selection (PS), ${\text{cov}}_{{p(d_{1} ,d_{2} )}}$, is obtained similarly and expressed as: ${\text{cov}}_{{p(d_{1} ,d_{2} )}} = \sigma_{{GCA_{d} }}^{2} \times r_{{MG_{d} }}^{2}$.

The phenotypic covariance between trait $k$ in the first stage (GS) and trait $d$ in the second stage (PS) is obtained from the correlation proposed by Dekkers (2007) as follows:

25 $$corr_{{p(k_{1} ,d_{2} )}} = r_{{MG_{k} }} \times h_{d} \times corr_{GCA(k,d)}$$

Expressing the correlations and the heritability as variances and covariances:

$$\frac{{{\text{cov}}_{{p(k_{1} ,d_{2} )}} }}{{\sqrt {\sigma_{{pk_{1} }}^{2} \times \sigma_{{pk_{2} }}^{2} } }} = r_{{MG_{k} }} \times \frac{{\sigma_{{GCA_{d} }} }}{{\sigma_{{pd_{2} }} }} \times \frac{{{\text{cov}}_{GCA(k,d)} }}{{\sqrt {\sigma_{{GCA(k_{1} )}}^{2} \times \sigma_{{GCA(d_{2} )}}^{2} } }}$$, yields

$${\text{cov}}_{{p(k_{1} ,d_{2} )}} = r_{{MG_{k} }} \times \frac{{\sigma_{{GCA_{d} }} }}{{\sigma_{{pd_{2} }} }} \times \frac{{{\text{cov}}_{GCA(k,d)} }}{{\sqrt {\sigma_{{GCA(k_{1} )}}^{2} \times \sigma_{{GCA(d_{2} )}}^{2} } }} \times \sigma_{{p_{{k_{1} }} }} \times \sigma_{{p_{{d_{2} }} }}$$

Canceling out the terms $\sigma_{{GCA_{d} }}$ and $\sigma_{{p_{{d_{2} }} }}$ yields ${\text{cov}}_{{p(K_{1} ,d_{2} )}} = r_{{MG_{k} }} \times \frac{{{\text{cov}}_{{GCA_{(k,d)} }} }}{{\sqrt {\sigma_{{GCAk_{1} }}^{2} } }} \times \sigma_{{pk_{1} }}$ and replacing the $\sigma_{{p_{{k_{1} }} }}$ = $\sigma_{{MEBV_{k} }}$ by $r_{{MG_{{k_{1} }} }} \times \sigma_{{GCA_{{k_{1} }} }}$ as explained in section (*iii)* yields:

26 $${\text{cov}}_{{p(K_{1} ,d_{2} )}} = r_{{MG_{k} }} \times \frac{{{\text{cov}}_{{GCA_{(k,d)} }} }}{{\sqrt {\sigma_{{GCAk_{1} }}^{2} } }} \times r_{{MG_{k1} }} \times \sigma_{{GCA_{{k_{1} }} }} = r_{{MG_{k} }}^{2} \times {\text{cov}}_{{GCA_{(k,d)} }}$$

Likewise, we obtain for the phenotypic covariance between trait $d$ in the first stage (GS) and trait $k$ in the second stage (PS):${\text{cov}}_{{p(d_{1} ,k_{2} )}} = r_{{MG_{d} }}^{2} \times {\text{cov}}_{{GCA_{(k,d)} }}$.

Finally, we organize the above-mentioned variances and covariances in a matrix ${\varvec{W}}$ as shown in Eq. (15) and the covariance between the phenotypic indices in the two stages (first GS and second PS) is obtained as shown in Eq. (16). As mentioned before, in the stage of GS, the phenotypic weights ${\varvec{b}}_{1}$ correspond to the economic weights ${\varvec{a}}$ so the covariance between phenotypic indices in the two stages can be expressed as:

27 $$cov_{{\left( {I_{1} ,I_{2} } \right)}} = {\varvec{a}}^{\prime}{\varvec{Wb}}_{2}$$

When the breeding strategy includes one stage of genomic selection followed by two stages of phenotypic selection, the variances of the phenotypic indices and covariances between phenotypic indices can be obtained by using the deductions of sections (*i)* to (*iv)*.

#### (v) Implementation of base and restricted index

If reliable estimates of phenotypic and genotypic parameters are lacking, the base index can be used for simultaneous improvement of two traits. In this situation, the index is calculated by summing up the weighted phenotypic values so that ${\varvec{b}} = {\varvec{a}}$. The computations to obtain the five covariances $\sigma_{{I_{1} }}^{2}$, $\sigma_{{I_{2} }}^{2}$, $cov_{{\left( {I_{1} ,H} \right)}} , { }cov_{{\left( {I_{2} ,H} \right)}}$ and $cov_{{\left( {I_{1} ,I_{2} ,} \right)}}$ required for the computation of the selection gain are performed as described in sections (*i)* to (*iv)*.

The restricted index was introduced by Kempthorne and Nordskog (1959) to find phenotypic weights so that a subset of traits is genetically improved while other traits remain unchanged, i.e., show null selection gain. For the case of phenotypic selection and assuming that trait $k$ is the trait to be improved, the phenotypic weights can be obtained as follows: (Wricke and Weber 1986)

28 $$b = \left[ {I - P^{ - 1} g_{k} [\left( {g_{k} ^{\prime}P^{ - 1} g_{k} } \right]^{ - 1} g_{k} ^{\prime} } \right] \times P^{ - 1} Ga$$

where ${\varvec{I}}$ is a 2 × 2 identity matrix, ${\varvec{g}}_{{\varvec{k}}}$ is the column of the matrix ${\varvec{G}}$ containing the variances and covariances for trait $k$. In the case of genomic selection, which implies that the matrices ${\varvec{G}}_{{{\varvec{MEBV}}}}$ and ${\varvec{P}}_{{{\varvec{MEBV}}}}$ are equal so that ${\varvec{P}}^{ - 1} {\varvec{G}}$ = ${\varvec{I}}$**,** the formula simplifies to

29 $$b~ = \left[ {I - P^{{ - 1}} ~g_{k} ~~[\left( {g_{k} 'P^{{ - 1}} g_{k} } \right]^{{ - 1}} ~g_{k} '} \right] \times ~a$$

The five covariances $\sigma_{{I_{1} }}^{2}$, $\sigma_{{I_{2} }}^{2}$, $cov_{{\left( {I_{1} ,H} \right)}} , { }cov_{{\left( {I_{2} ,H} \right)}}$ and $cov_{{\left( {I_{1} ,I_{2} ,} \right)}}$ required for the computation of the selection gain can be obtained as described in sections (*i)* to (*iv)*.

#### (vi) Implementation of Independent Culling Levels ICL

For the implementation of ICL in strategy *GSrapid*, the following variances and covariances were computed, assigned to a variance–covariance matrix, and transformed to a correlation matrix as required by the package *selectiongain*. One matrix was constructed for each independent trait. The annual selection gain *ΔG*_*a*_ was obtained using the covariance matrix and the selected fractions for trait 1 and 2 (${\alpha }_{1}$ and ${\alpha }_{2}$), with restriction ${\alpha }_{1}\times {\alpha }_{2}=\alpha$ (Wricke and Weber 1986). There, $\alpha$ corresponds to the total selected fraction at the respective stage in the optimum allocation of resources of the optimum index.

1. Phenotypic variance for trait *k* at the phenotypic selection stage: see Eq. (3).
2. Phenotypic covariance between traits at the same selection stage: see Eq. (4).
3. The variance of MEBV for trait *k:* see Eq. (18).
4. The covariance between MEBV of two traits (*k* and *d*): see Eq. (19).
5. The covariance of MEBV of trait *k* and phenotypes of trait *k*: see Eq. (24).
6. The covariance of MEBV of trait *k* and phenotypes of trait *d*: see Eq. (26).

For the implementation of ICL in strategy *PSstandard*, the same procedure was applied by calculating the variances and covariances as follows:

1. Phenotypic variance for trait *k* or *d* at phenotypic selection stages 1 or 2: see Eq. (3).
2. Phenotypic covariance between traits *k* and *d* at phenotypic selection stages 1 or 2: see Eq. (4).
3. Phenotypic covariance across stages for trait *k* or *d*: see Eq. (13).
4. Phenotypic covariance between traits *k* and *d* across stages 1 and 2: see Eq. (14).

## Declarations

### Availability of data and material

The datasets generated during and/or analyzed during the current study are available from the corresponding author on reasonable request.

### Code availability

The open-source R package “*selectiongain*” version 2.0.65 is online available at https://cran.r-project.org/web/packages/selectiongain/index.html

## References

[CR1] Albrecht T, Auinger H-J, Wimmer V (2014). Genome-based prediction of maize hybrid performance across genetic groups, testers, locations, and years. Theor Appl Genet.

[CR2] Baker RJ (1986). Selection indices in plant breeding.

[CR3] Bernardo R (2010). Breeding for quantitative traits in plants.

[CR4] Ceron-Rojas JJ, Crossa J, Arief VN, et al (2015) A Genomic Selection Index Applied to Simulated and Real Data. G3 (Bethesda) 5: 2155–64 . doi: 10.1534/g3.115.019869 10.1534/g3.115.019869 PMC4592997 26290571

[CR5] Cochran WG (1951) Improvement by means of selection. In: Proceedings of the Second Berkeley Symposium on Mathematical Statistics and Probability. University of California, pp 449–470

[CR6] Cole JB, VanRaden PM (2017). Possibilities in an age of genomics: The future of selection indices. J Dairy Sci.

[CR7] Dekkers JCM (2007). Prediction of response to marker-assisted and genomic selection using selection index theory. J Anim Breed Genet.

[CR8] Eagles HA, Frey KJ (1974). expected and actual gains in economic value of oat lines from five selection methods. Crop Sci.

[CR9] Elgin JH, Hill RR, Zeiders KE (1970) Comparison of Four Methods of Multiple Trait Selection for Five Traits in Alfalfa. Crop Sci 190–193

[CR10] Gaynor RC, Gorjanc G, Bentley AR (2017). A two-part strategy for using genomic selection to develop inbred lines. Crop Sci.

[CR11] Goddard ME, Hayes BJ, Meuwissen THE (2011). Using the genomic relationship matrix to predict the accuracy of genomic selection. J Anim Breed Genet.

[CR12] Graffius R (1964). A geometry for plant breeding. Crop Sci.

[CR13] Grieder C, Dhillon BS, Schipprack W, Melchinger AE (2012) Breeding maize as biogas substrate in Central Europe: I. Quantitative-genetic parameters for testcross performance. Theor Appl … 124:971–980 . doi: 10.1007/s00122-011-1761-y 10.1007/s00122-011-1761-y 22159756

[CR14] Hayes BJ, Panozzo J, Walker CK (2017). Accelerating wheat breeding for end-use quality with multi-trait genomic predictions incorporating near infrared and nuclear magnetic resonance-derived phenotypes. Theor Appl Genet.

[CR15] Hazel LN (1943). The Genetic Basis for Constructing Selection Indexes. Genetics.

[CR16] Hazel I, Lush JL (1942). The efficiency of three methods of selection. J Hered.

[CR17] Heslot N, Jannink J-L, Sorrells ME (2015). Perspectives for genomic selection applications and research in plants. Crop Sci.

[CR18] Kamenya SN, Mikwa EO, Song B, Odeny DA (2021). Genetics and breeding for climate change in Orphan crops. Theor Appl Genet.

[CR19] Kempthorne O, Nordskog AW (1959). Restricted selection indices. Biometrics.

[CR20] Laidig F, Piepho H-P, Rentel D (2016). Breeding progress, environmental variation and correlation of winter wheat yield and quality traits in German official variety trials and on-farm during 1983–2014. Theor Appl Genet.

[CR21] Liu G, Zhao Y, Gowda M (2016). Predicting Hybrid Performances for quality traits through genomic-assisted approaches in central european wheat. PLoS ONE.

[CR22] Longin CFH, Gowda M, Mühleisen J (2013). Hybrid wheat: quantitative genetic parameters and consequences for the design of breeding programs. Theor Appl Genet.

[CR23] Longin CFH, Sieber AN, Reif JC (2013). Combining frost tolerance, high grain yield and good pasta quality in durum wheat. Plant Breed.

[CR24] Longin CFH, Mi X, Würschum T (2015). Genomic selection in wheat: optimum allocation of test resources and comparison of breeding strategies for line and hybrid breeding. Theor Appl Genet.

[CR25] Marulanda JJ, Mi X, Melchinger AE (2016). Optimum breeding strategies using genomic selection for hybrid breeding in wheat, maize, rye, barley, rice and triticale. Theor Appl Genet.

[CR26] Melchinger AE, Longin CFH, Utz HF, Reif JC (2005) Hybrid maize breeding with doubled haploid lines: quantitative genetic and selection theory for optimum allocation of resources. In: Proceedings of the 41st annual Illinois corn breeders school. Urbana-Champaign, pp 8–21

[CR27] Meuwissen THE, Hayes BJ, Goddard ME (2001). Prediction of Total Genetic Value Using Genome-Wide Dense Marker Maps. Genetics.

[CR28] Mi X, Utz HF, Technow F, Melchinger AE (2014). Optimizing resource allocation for multistage selection in plant breeding with R package selectiongain. Crop Sci.

[CR29] Mi X, Utz HF, Melchinger AE (2015). Selectiongain: an R package for optimizing multi-stage selection. Comput Stat.

[CR30] Michel S, Ametz C, Gungor H (2016). Genomic selection across multiple breeding cycles in applied bread wheat breeding. Theor Appl Genet.

[CR31] Michel S, Löschenberger F, Ametz C (2019). Simultaneous selection for grain yield and protein content in genomics - assisted wheat breeding. Theor Appl Genet.

[CR32] Mistele M, Zeddies J, Urz HF, Melchinger AE (1994). Economic aspects of breeding for yield and quality traits in forage maize. I. Determination of Economic Weights Plant Breed.

[CR33] Osthushenrich T, Frisch M, Zenke-Philippi C (2018). Prediction of means and variances of crosses with genome-wide marker effects in barley. Front Plant Sci.

[CR34] Pesek J, Baker R (1969). Comparison of tandem and index selection in the modified pedigree method of breeding self-pollinated species. Canad Jour Plant Sci.

[CR35] R Core Team (2016) R: A language and environment for statistical computing

[CR36] Rapp M, Lein V, Lacoudre F (2018). Simultaneous improvement of grain yield and protein content in durum wheat by different phenotypic indices and genomic selection. Theor Appl Genet.

[CR37] Rosielle AA, Frey KJ (1975). Application of Restricted Selection Indices for Grain Yield Improvements in Oats. Crop Sci.

[CR38] Smith HF (1936). A discriminant function for plant selection. Ann Eugen.

[CR39] Tallis GM (1961). The moment generating function of the truncated multi-normal distribution. J R Stat Soc Ser B.

[CR40] Thorwarth P, Piepho HP, Zhao Y, et al (2018) Higher grain yield and higher grain protein deviation underline the potential of hybrid wheat for a sustainable agriculture. doi: 10.1111/pbr.12588

[CR41] Utz HF (1969). Mehrstufenselektion in der Pflanzenzüchtung (In German).

[CR42] Vinson WE (1971) The use of independent culling levels and selection index procedures in selecting future sires for artificial insemination. Retrospective Thesis and Dissertations 4518. Iowa State University.

[CR43] Williams JS (1962). The evaluation of a selection index. Biometrics.

[CR44] Wricke G, Weber E (1986). Quantitative Genetics and Selection in Plant Breeding.

[CR45] Young SSY (1961). A further examination of the relative efficiency of three methods of selection for genetic gains under less-restricted conditions. Genet Res.

[CR46] Zhao Y, Zeng J, Fernando R, Reif JC (2013). Genomic prediction of hybrid wheat performance. Crop Sci.

