# Supplementary material for: Optimum breeding strategies using genomic and phenotypic selection for the simultaneous improvement of two traits
Source: Theor Appl Genet. 2021 Oct 7;134(12):4025–42. doi: 10.1007/s00122-021-03945-5 (PMC8580912; doi:10.1007/s00122-021-03945-5)
Supplement: Supplementary file 1 — Supplementary file1 (DOCX 14 kb) [file 122_2021_3945_MOESM1_ESM.docx]

**Suppl. Table 1** Scenarios and goals for the simultaneous improvement of two traits for the practical section on hybrid wheat. (GY = grain yield, Prot = grain protein content, SDS = sedimentation volume)

|  |  | Economic weight | | Variance components for GY | | | | | Variance components for Prot or SDS | | | | | Covariance |
| --- | --- | --- | --- | --- | --- | --- | --- | --- | --- | --- | --- | --- | --- | --- |
| **Goal** | *ρ* _Genetic_ | GY | Prot or SDS | $\sigma_{GCA}^{2}$ | $\sigma_{GCA\times L}^{2}$ | $\sigma_{SCA}^{2}$ | $\sigma_{SCA\times L}^{2}$ | $\sigma_{error}^{2}$ | $\sigma_{GCA}^{2}$ | $\sigma_{GCA\times L}^{2}$ | $\sigma_{SCA}^{2}$ | $\sigma_{SCA\times L}^{2}$ | $\sigma_{error}^{2}$ | ${COV}_{GCA}$ |
| 1. GY and Prot | -0.4 | 1 | 0.12 | 5.7 | 5.19 | 1.88 | 2.94 | 24.37 | 0.07 | 0.03 | 0.01 | 0.00* | 0.09 | -0.25 |
| 2. GY and SDS | -0.18 | 1 | 0.12 | 5.7 | 5.19 | 1.88 | 2.94 | 24.37 | 23.54 | 1.58 | 0.64 | 0.25 | 6.99 | -2.09 |

*Although variance component $\sigma_{SCA\times L}^{2}$ for protein was not equal to zero, it was too small to be considered in our model calculations.
